# Editorial: Global perspectives on genetic diagnosis and treatments for inherited retinal diseases

**DOI:** 10.3389/fopht.2026.1943908

**Published:** 2026-08-25

**Authors:** Yusuke Murakami, Pei-Kang Liu, Dyah Ayu Windy, Arjun Shrestha

**Affiliations:** 1 Department of Ophthalmology, Graduate School of Medical Sciences, Kyushu University, Fukuoka, Japan; 2 Department of Ophthalmology, Kaohsiung Medical University, Kaohsiung, Taiwan; 3 Department of Ophthalmology, Hasanuddin University, Makassar, Indonesia; 4 BP Eye Foundation, Hospital for Children, Eye, ENT and Rehabilitation Services, Kathmandu, Nepal

**Keywords:** genotype-phenotype, inherited retinal degeneration (IRD), PGT-M, precision medicine, retinitis pigmentosa

Inherited retinal diseases (IRDs) comprise a heterogeneous group of inherited disorders characterized by progressive degeneration of photoreceptors and the retinal pigment epithelium, ultimately leading to irreversible visual impairment. More than 300 causative genes have been identified, and remarkable advances in next-generation sequencing, gene therapy, and genome engineering have transformed the landscape of IRD research over the past decade. Nevertheless, these advances have not been distributed evenly across the world. Most large genetic studies, natural history cohorts, and interventional trials have originated from North America and Europe, whereas many regions continue to face limited access to genetic diagnosis and emerging therapies.

This Research Topic, *Global Perspectives on Genetic Diagnosis and Treatments for Inherited Retinal Diseases*, was launched to encourage contributions from diverse geographic regions and to highlight the expanding global efforts toward understanding and treating IRDs. The eight articles collected in this Topic reflect the remarkable breadth of contemporary IRD research, ranging from clinical genetics and deep phenotyping to immunopathology, reproductive genetics, and therapeutic perspectives. Together, they illustrate how advances in molecular diagnosis are increasingly being translated into individualized patient care.

A major theme emerging from this Research Topic is the continuing evolution of genetic diagnosis and genotype–phenotype correlation. Several studies demonstrate that identifying pathogenic variants alone is often insufficient; careful clinical phenotyping remains essential. Sakai et al. proposed a novel phenotypic classification of *EYS*-associated retinopathy based on visual impairment patterns, revealing that pericentral disease is more common than previously appreciated and frequently accompanied by early macular dysfunction. Their work demonstrates the value of integrating visual field characteristics into genotype-based disease classification. Similarly, Hirakata et al. described remarkable intrafamilial phenotypic variability associated with *CDHR1* variants within a single Japanese family, emphasizing the complexity of variant interpretation and the importance of cautious genetic counseling. Duan et al. further expanded the phenotypic spectrum of *MYO7A* by reporting fluctuating cystoid macular edema in patients lacking the classic syndromic manifestations of Usher syndrome, reminding clinicians that inherited retinal diseases continue to reveal unexpected clinical presentations.

Another important message emerging from this Research Topic is that genetic diagnosis remains an evolving process. Koyanagi et al. analyzed more than 500 patients with retinitis pigmentosa and demonstrated that the diagnostic yield of current targeted sequencing panels declines markedly with increasing patient age. Their findings suggest that elderly patients may harbor undiscovered disease-causing variants or genetic mechanisms that remain beyond the reach of existing diagnostic approaches, underscoring the need for continued gene discovery and refinement of sequencing strategies.

Beyond diagnosis, several articles illustrates how advances in molecular research are shaping future clinical care. Tao et al. identified an IL-23-related inflammatory network associated with cystoid macular edema in retinitis pigmentosa, supporting neuroinflammation as a potential therapeutic target beyond gene replacement. Complementing this work, Suleman provides a comprehensive review of current therapeutic strategies for retinitis pigmentosa, including gene and cell therapies, optogenetics, retinal prostheses, pharmacological interventions, and neuroprotective approaches.

The Research Topic also illustrates how genetic technologies are expanding into clinical practice. Li et al. demonstrated the successful use of preimplantation genetic testing (PGT-M) to prevent transmission of an RS1 mutation causing X-linked retinoschisis, emphasizing the growing role of genomic medicine in family planning. Although outside the traditional spectrum of IRDs, He et al. reported a novel RB1 splice-site mutation in familial retinoblastoma, further emphasizing the value of molecular diagnosis in inherited ocular diseases.

**Figure 1:**
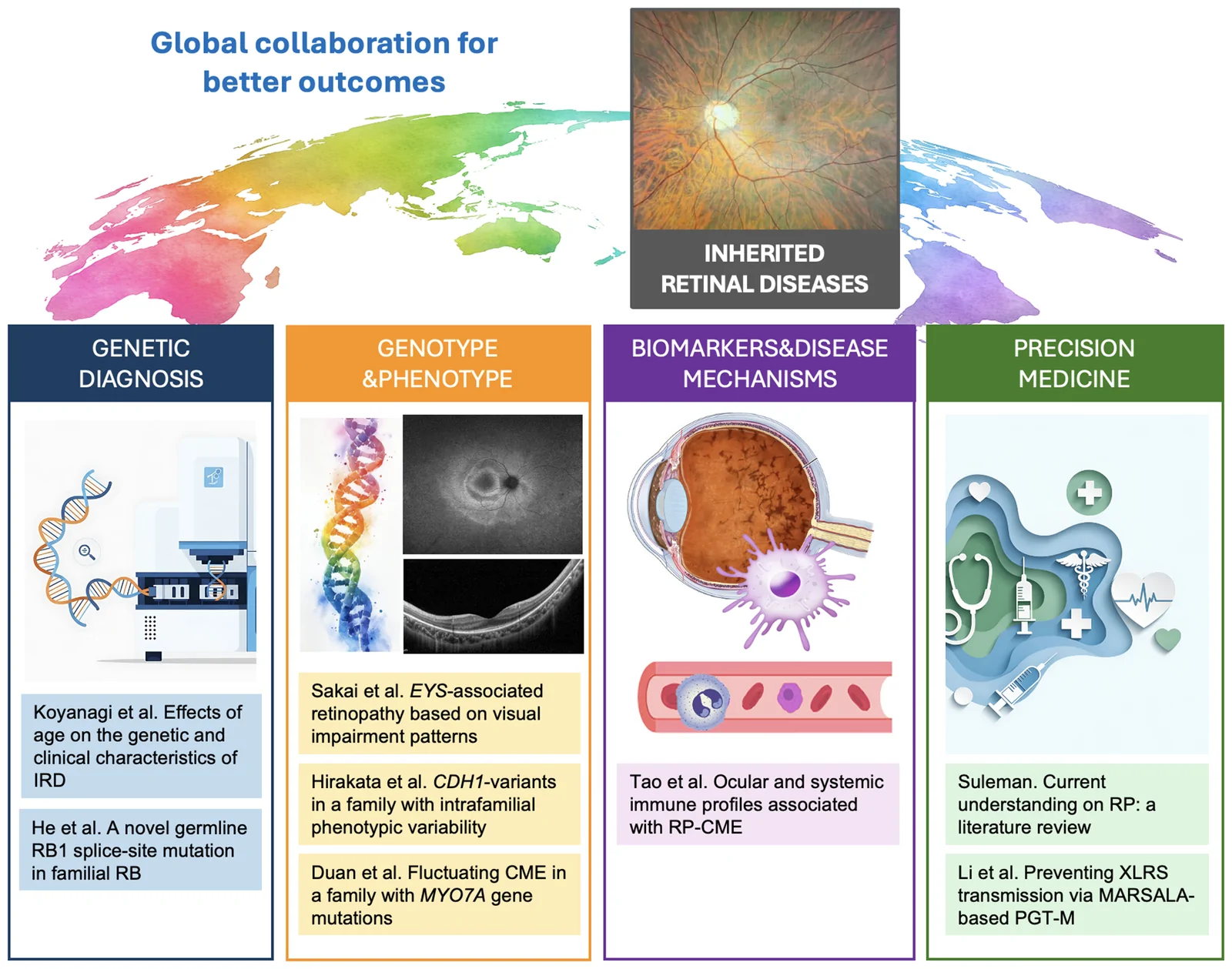
Global perspectives on genetic diagnosis and treatment for inherited retinal diseases.

Finally, this Research Topic illustrates the importance of a global perspective in IRD research. Contributions from Japan, China, and Indonesia highlight regional genetic diversity and clinical variability, which will become increasingly important as precision medicine and gene-specific therapies continue to expand (**Figure 1**). However, this Research Topic includes no articles from Africa. Given the substantial genetic diversity within and among African populations, further studies involving African cohorts are essential to ensure equitable advances in IRD diagnosis and treatment.

Taken together, these studies demonstrate that progress in IRD research relies on integrating molecular genetics, deep phenotyping, biomarker discovery, and translational research. We thank all authors, reviewers, and the editorial staff of *Frontiers in Ophthalmology* for their valuable contributions. We hope this Research Topic will promote further international collaboration and accelerate the development of more precise diagnostics and effective treatments for patients with inherited retinal diseases worldwide.

